# Atrial Fibrillation, Atrial Myopathy, and Thromboembolism: The Additive Value of Echocardiography and Possible New Horizons for Risk Stratification

**DOI:** 10.3390/jcm13133921

**Published:** 2024-07-04

**Authors:** Alessandro Campora, Matteo Lisi, Maria Concetta Pastore, Giulia Elena Mandoli, Yu Fu Ferrari Chen, Annalisa Pasquini, Andrea Rubboli, Michael Y. Henein, Matteo Cameli

**Affiliations:** 1 Department of Medical Biotechnologies, Division of Cardiology, University of Siena, Viale Bracci 1, 53100 Siena, Italy; pastore2411@gmail.com (M.C.P.); giulia_elena@hotmail.it (G.E.M.); matteo.cameli@unisi.it (M.C.); 2 Department of Emergency, Internal Medicine and Cardiology—AUSL Romagna, Division of Cardiology, Ospedale S. Maria delle Croci, Viale Randi 5, 48121 Ravenna, Italy; matteo.lisi@hotmail.it (M.L.); andrea.rubboli@auslromagna.it (A.R.); 3 Cardiovascular Division, Pisa University Hospital and University of Pisa, Via Paradisa 2, 56124 Pisa, Italy; y.ferrarichen@studenti.unipi.it; 4 Department of Cardiovascular and Thoracic Sciences, Fondazione Policlinico Universitario A. Gemelli IRCCS, Università Cattolica del Sacro Cuore, 00168 Rome, Italy; annalisa.pasquini@policlinicogemelli.it; 5 Department of Public Health and Clinical Medicine, Umeå University, 90187 Umeå, Sweden; henein@gmail.com

**Keywords:** atrial fibrillation, atrial myopathy, left atrial strain

## Abstract

Atrial fibrillation (AF) is the most common cardiac sustained arrhythmia, and it is associated with increased stroke and dementia risk. While the established paradigm attributes these complications to blood stasis within the atria and subsequent thrombus formation with cerebral embolization, recent evidence suggests that atrial myopathy (AM) may play a key role. AM is characterized by structural and functional abnormalities of the atria, and can occur with or without AF. Moving beyond classifications based solely on episode duration, the 4S-AF characterization has offered a more comprehensive approach, incorporating patient’s stroke risk, symptom severity, AF burden, and substrate assessment (including AM) for tailored treatment decisions. The “ABC” pathway emphasizes anticoagulation, symptom control, and cardiovascular risk modification and emerging evidence suggests broader benefits of early rhythm control strategies, potentially reducing stroke and dementia risk and improving clinical outcomes. However, a better integration of AM assessment into the current framework holds promise for further personalizing AF management and optimizing patient outcomes. This review explores the emerging concept of AM and its potential role as a risk factor for stroke and dementia and in AF patients’ management strategies, highlighting the limitations of current risk stratification methods, like the CHA2DS2-VASc score. Echocardiography, particularly left atrial (LA) strain analysis, has shown to be a promising non-invasive tool for AM evaluation and recent studies suggest that LA strain analysis may be a more sensitive risk stratifier for thromboembolic events than AF itself, with some studies showing a stronger association between LA strain and thromboembolic events compared to traditional risk factors. Integrating it into routine clinical practice could improve patient management and targeted therapies for AF and potentially other thromboembolic events. Future studies are needed to explore the efficacy and safety of anticoagulation in AM patients with and without AF and to refine the diagnostic criteria for AM.

## 1. Introduction

Atrial fibrillation (AF) is the most common sustained cardiac arrhythmia among adults, and it is associated with significant morbidity and mortality, including increased risk of stroke and dementia. While the established paradigm attributes these complications to blood stasis within the atria and subsequent thrombus formation with cerebral embolization, recent evidence suggests a key role for atrial myopathy (AM).

AM is characterized by structural and functional abnormalities of the atria, which can occur with or without AF. The study of left atrial (LA) deformation with 2D speckle tracking echocardiography, which measures the ability of the LA to contract and relax, has demonstrated high sensitivity and specificity in identifying myocardial fibrosis and increased atrial stiffness, both markers of AM. Recently, different studies have shown that LA strain can be used to stratify the risk of stroke and dementia, even in patients who do not have AF, suggesting that LA strain alterations, reflecting the presence of underlying AM, may be a more sensitive risk stratifier of thromboembolic events than the presence of AF itself. By exploring these aspects, this article aims to provide a comprehensive understanding of the emerging role of AM in AF-related complications and its potential to refine risk stratification and patient management strategies.

## 2. Atrial Fibrillation: The Size of the Problem

AF is a supraventricular tachyarrhythmia characterized by an incoordinate atrial electrical activation and consequently ineffective atrial contraction. Electrocardiographic (ECG) characteristics of AF include:

- irregular R-R intervals (when atrioventricular conduction is not impaired);
- absence of distinct repeating P waves;
- irregular atrial activations [1].

AF is the most frequently sustained cardiac arrhythmia among adults [2], with estimated prevalence of 0.4–1% in the general population and increasing with age, affecting 9% of people above 80 years [3]. The symptoms associated with AF are heterogeneous, as it may vary from asymptomatic AF incidentally diagnosed during a routine ECG, to a highly symptomatic form, which manifests through chest pain, shortness of breath, syncope, palpitations, or even stroke or transient ischemic attack (TIA). A diagnosis of AF is associated with a 1.5- to 2-fold increased risk of all-cause mortality and increased morbidity in the general population [4] and diminished quality of life [5], particularly due to higher risk of ischemic stroke and dementia [6].

## 3. Pathophysiological Pathway of Atrial Fibrillation

Significant efforts have been made over the years to define the underlying cellular, molecular, and electrophysiological changes that predispose patients to the occurrence and maintenance of AF [7]. Progress has been limited by the understanding that AF is a complex arrhythmia that can be the final result of various different pathophysiological processes, with significant heterogeneity between patients [7]. Age is a prominent AF risk factor, but there is an increasing burden of other comorbidities including hypertension, diabetes mellitus, heart failure (HF), coronary artery disease (CAD), chronic kidney disease [8], obesity, and obstructive sleep apnea [9,10,11,12,13], which are all potent contributors to AF development and progression to permanent AF [14,15].

The development of AF and its lifetime risk depends on age, genetic, and (sub)clinical factors [16,17,18]. The impact of clinical risk factor burden and multiple comorbidities on AF suggests that an early intervention and optimum risk factor control could reduce the incidence of AF.

The mechanism that these risk factors potentially share is the capability to induce atrial electrical and structural remodeling. Electrical remodeling encompasses changes in the properties of ion channels affecting atrial myocardial depolarization and conduction, while structural remodeling refers to alterations in the tissue architecture, both microscopic (e.g., myocardial fibrosis) and macroscopic (e.g., atrial dilation). At this point, the initiation and maintenance of AF can be linked to the interaction between a trigger and the substrate. A ‘trigger’ is a rapidly firing focus that can act as an initiator of the arrhythmia, the maintenance of which generally requires a ‘remodeled substrate’, that is, altered electrophysiological, mechanical, and anatomical characteristics of the atria that sustain AF. 

It is thought that there is a progression over time from a trigger-driven disease, through to development of an atrial function substrate, followed by predominant atrial structural remodeling [8]. This would correspond to the clinical observation that AF is often initially paroxysmal, before progressing to a persistent and ultimately permanent form of arrhythmia [19].

## 4. Association between AF, Stroke/TIA, and Dementia—The Emerging Concept of Atrial Myopathy

The presence of AF is associated with a fivefold increased risk of stroke; in fact, TIA or ischemic stroke represents the first manifestation of AF in 2–5% of patients [6]. This is usually attributed to LA appendage thrombi formation as a result of blood stasis, with subsequent clot dislodgement and embolization to the systemic circulation, more often to the brain, and frequently after restoration of sinus rhythm. 

Furthermore, an independent correlation between AF and various forms of dementia, including Alzheimer disease, has been observed in the AF population, irrespective of stroke occurrence [20,21]. The pathophysiology is thus likely to be multifactorial, which has not been fully explained [20]. One plausible mechanism may be repetitive microclot/macroclot embolization, chronically leading to brain dysfunction. Studies supporting this hypothesis showed a reduction in the incidence of dementia in AF patients treated with optimum anticoagulation [22,23], or in patients undergoing effective and early AF ablation [24,25]. This would suggest a shared pathophysiology for dementia and stroke/TIA as connected to the atrial disease. The complexity of the mechanism by which these morbidities result from AF has yet to be completely understood, in particular, the interaction of various factors involved in thrombus formation, which should allow better clinical risk stratification and optimum targeted therapies in these patients. 

The well-established increased risk of thrombus formation stems from an alteration of the normal hemostatic mechanisms, encapsulated in the concept of Virchow’s triad. This triad comprises three key elements: alterations in blood constituents (including platelet factor 4, von Willebrand factor, fibrinogen, β-thromboglobulin, and D-dimer), the presence of blood vessel wall diseases, and reduced blood flow. Notably, the onset of AF has been demonstrated to be associated with all three components of Virchow’s triad. Specifically, with the increased activation of the coagulation cascade and platelet reactivity and with impaired fibrinolysis, processes are also amplified by the usually pre-existing comorbidities. The presence of atrial fibrosis and endothelial dysfunction is related to the development of AF, which promotes further atrial remodeling, thereby providing an increased risk for clot formation and subsequent embolization. In addition, it is also well demonstrated that LA dilation and the loss of atrial contractile function reduce blood flow, specifically in the LA appendage, as it has been shown with various imaging techniques [26]. For years, the atrial blood stasis hypothesis has been acknowledged as the mechanism of AF-related thromboembolism and morbidity [27]; however, recent evidence has emerged to suggest the presence of atrial myopathy (AM) as an alternative hypothesis.

AM (or atrial cardiomyopathy or atrial cardiopathy) is defined as “any complex structural, architectural, contractile or electrophysiological changes affecting the atria with the potential for producing clinically relevant manifestations” and appears as LA dysfunction and dilation [28,29]. The working group between the European Heart Rhythm Association (EHRA), the Heart Rhythm Society (HRS), the Asian Pacific Heart Rhythm Society (APHRS), and Sociedad Latino Americana de Estimulacion Cardiaca y Electrofisiologia (SOLAECE) proposed in 2016 a working histological/pathophysiological classification scheme for AM (Table 1), which may help to convey the primary underlying pathology that led to its development. Emerging evidence suggests that thromboembolism can occur in the setting of AM even in the absence of AF [30]. Elevated systemic inflammatory status associated with various comorbidities or the presence of AF itself, and the subsequent fibrotic process of LA wall, are primary determinants of the loss of conduit function of the atrial chamber, even in the absence of AF. Furthermore, inflammation and fibrosis may directly enhance the thrombogenicity of the atrial endocardium. However, AM and AF are strictly correlated with a cause–consequence relationship. This relationship is based on the close association between atrial fibrosis, one of the main characteristics of AM, and AF. Even a microscopic scar has been documented to affect LA compliance and mechanical function, leading to the development of AF [31]. Furthermore, AF and the subsequent volume and fluid overload may themselves cause atrial remodeling, increasing atrial wall stiffness and fibrosis, and therefore provoking or worsening AM [32].

## 5. Evaluation of Atrial Cardiomyopathy

The diagnosis of AM could be suggested based on ECG findings, as AF or other atrial arrhythmias [33,34], serum biomarkers, and imaging evidence for LA dilation and dysfunction, often associated with myocardial fibrosis [28,35,36]. 

LA dilation, in the absence of mitral valve disease, is usually an indicator of chronic increase in wall tension, commonly due to increased LA pressure [37,38,39,40], paired with impairment of LA function related to AM [41,42]. LA dilation has been shown to be related to increased incidence of AF and stroke [43,44,45,46,47,48,49,50,51,52], increased risk of overall mortality after myocardial infarction [41,42,53,54], increased risk of death and hospitalization in patients with dilated cardiomyopathy [55,56,57,58,59,60,61,62,63], as well as major cardiac events or death in patients with diabetes mellitus [64].

LA dilation is also a well-known marker of severity and chronicity of diastolic dysfunction and its consequent raised LA pressure [37,38,39,40]. Despite that, Kojima T et al. [65] and others have shown that LA function deteriorates before cavity enlargement, with 36% of patients with AF having normal LA size [66]. Such controversy suggests the need for thorough and comprehensive assessment of atrial anatomy, structure, and function for accurate diagnosis of AM. 

Evaluation of atrial morphology is typically carried out with 2D or 3D echocardiography. For assessment of atrial size, the parasternal long-axis linear dimension using M-mode is the most commonly used method [44,45,46,47,48,49,50,51,52,53,54,55,56,57,58,64,67,68,69]. However, considering the complex 3D nature of the atrium and the usually non-uniform atrial remodeling, this measurement does not guarantee an accurate reflection of LA size [70,71,72,73,74]. Hence, measurement of LA volume has emerged as a more accurate prognostic indicator in a variety of cardiac diseases [49,51,53,54,55,56,57,58,64,67,68,69,70,71,72,73,74,75,76,77,78,79,80]. LA volume from 2D images is best measured using the disk summation algorithm since it includes fewer geometric assumptions [81,82]. Two-dimensional echocardiographic LA volumes are typically smaller than those reported from computed tomography (CT) or cardiac magnetic resonance imaging (CMR) [83,84,85,86,87]; however, LA volumes measured by three-dimensional echocardiography have been shown to correlate well with cardiac CT [88,89] and CMR [90,91], having shown a superior prognostic predictive power compared to two-dimensional LA volumes [92,93]. 

Overall, the recommended normal upper limit for indexed LA volume is 34 mL/m^2^ for both genders, which fits well with a risk-based approach for determining a cut-off between a normal and an enlarged LA [43,77,78,79].

LA function can be studied by analyzing the electrical remodeling process from surface 12 lead ECG (P wave terminal force in lead V1, P wave axis alterations, P wave voltage reduction, P wave increased area, P wave dispersion or prolonged duration, PR interval (interatrial conduction block)) [94], or more precisely during an electrophysiological study. 

Routine conventional echocardiographic indexes used for studying LA function have limitations, but the evaluation of longitudinal deformation by speckle tracking echocardiography has proven to be a very reliable parameter. LA function has been studied with different echocardiographic tools, including pulsed-wave Doppler measurements of late (mitral A) diastolic filling and pulmonary vein atrial reversal velocity, but their absolute values are affected by many factors, including age, reduced LV compliance, and loading conditions [39,95,96,97,98,99,100,101,102,103]. 

The time between the onset of surface ECG “P” wave and the onset of “a” wave on tissue Doppler imaging (TDI), or total atrial conduction time, can be used as a non-invasive marker of atrial electromechanical delay (AEMD) [104,105,106,107,108,109,110,111], which has shown to be an accurate, yet indirect marker of atrial function. This index measures the time required for atrial depolarization to occur, which results in active atrial contraction representing a more complete measure of the extent of atrial remodeling than other indices [112]. An increase in TDI-derived AEMD has been shown to predict AF recurrence in patients with paroxysmal AF [104], particularly if measured in the lateral leads, and has been shown to be non-inferior to LA volume index in identifying those patients [113]. 

A correlation between TDI-derived AEMD duration and the degree of right atrial appendage fibrosis has been demonstrated in a histological validation study by Müller et al. [114]. A good agreement has also been demonstrated between TDI-derived AEMD duration and total atrial conduction time measured in an electrophysiological study in healthy individuals [115]. Its duration was also shown to be affected by several risk factors which are known to play a significant role in atrial remodeling, including age, hypertension, valvular disease, LV diastolic dysfunction, sleep apnea, and increased body mass index [112]. Additionally, an increase in TDI-derived AEMD was found to be related to increased LA volume and inversely related to LA reservoir strain [116]. This index has been shown to be an independent predictor of AF after cardiac surgery [117], as well as a predictor of AF recurrence after electrical cardioversion [118] and catheter ablation [119]. In a recent prospective study of patients free of AF after successful catheter ablation who were not on anticoagulants, a prolonged TDI-derived AEMD was associated with increased risk of stroke incidence and has been demonstrated to improve the predictive performance of the CHA2DS2-VASc score, increasing the area under the ROC curve from 0.75 to 0.85 [120]. 

Two-dimensional speckle tracking echocardiography has emerged as a more sensitive marker for detecting early functional remodeling before anatomical alterations occur [121,122,123,124,125,126,127,128,129,130,131,132,133,134,135]. Strain and strain rate are two measures of myocardial deformation based on estimating spatial gradients in myocardial velocities [121,124,128,129,136,137,138,139,140]. This technique offers important information on early modification of LA structure and function, before volume changes, and is associated with the occurrence and persistence of AF [35,141]. Furthermore, patients with paroxysmal AF have been shown to have increased LA stiffness, which is described as low relaxation properties, one of the main features of AM that can be non-invasively measured as the ratio between E/e’ and LA strain [142].

Normality ranges for LA strain values in healthy individuals have been evaluated in various studies, with the NORRE study [143] and Pathan’s 2019 meta-analysis [144] being the most prominent (Table 2). 

Abnormalities in atrial strain have been observed in many conditions, including AF, valvular pathology, HF, hypertension, diabetes, and cardiomyopathies [124,125,131,132,133,134,135]. This is supported by findings from the MASCOT HIT study [145] involving patients with evidence of hypertension or aortic stenosis (LA pressure overload), mitral regurgitation, or HF (LA volume–pressure overload). The mean LA strain values in these groups were 23.0 ± 8.5% and 18.9 ± 9.2%, respectively.

In patients with history of paroxysmal or persistent AF, Lin et al. [146] reported mean LA strain values of 19.5 ± 9.6%, while those with persistent AF had values of 9.1 ± 3.1%. Cameli et al. [147] investigated asymptomatic patients with normal LA and LV dimensions and normal ejection fraction. They found that individuals with diabetes mellitus or hypertension had significantly reduced PALS compared to healthy controls. PALS values were 26.2 ± 7.1% in subjects with DM, 31.9 ± 10.3% in patients with hypertension, and 39.2 ± 8.7% in controls. PALS values were also significantly lower in patients with both comorbidities (20.4 ± 6.5). 

The presence of these early alterations in LA strain underscores the importance of echocardiographic assessment of atrial function. This approach can reveal the presence of early cardiac damage even in the absence of specific symptoms, thereby unmasking underlying AM and providing clinicians with a valuable tool for implementing early preventive strategies. 

Population-based studies have shown the prognostic value of LA strain analysis of long-term outcome [124,130]. Interestingly, LA dysfunction with changes in strain and strain rate has been observed in patients with amyloidosis even in the absence of other echocardiographic features of cardiac involvement, thus highlighting its possible application as an early marker of cardiac involvement [137]. The study of LA deformation with 2D speckle tracking echocardiography has also demonstrated high sensitivity in identifying myocardial fibrosis and increased cavity stiffness as compared to CMR measures [35], invasive electrophysiological studies with high-density voltage mapping [148], and with invasive biopsy assessment [149]. Considering the CMR’s high cost and the risk of side-effects from gadolinium, echocardiography has been proved as the modality of choice for screening and serially following patients with diseases involving LA morphology and function [67,150].

## 6. Potential Applications of AM Study in Clinical Practice

The management of AF has evolved beyond a simple classification based on episode duration and temporal patterns. This approach, which categorized AF as first-diagnosed, paroxysmal, persistent/long-standing persistent, and permanent, provided limited guidance for treatment strategies. Recently, a more comprehensive approach has been adopted, exemplified by the 4S-AF characterization, proposed by Potpara et al. [151]. This framework incorporates four key domains with significant treatment and prognostic implications:

- Stroke Risk: Assessed using the CHA2DS2-VASc score, this domain helps identify patients at high risk for stroke and recommend anticoagulation therapy.
- Symptom Improvement: Evaluated using the EHRA symptom score [152], this domain guides the selection of strategies to improve patient well-being, such as rate control or rhythm control.
- Severity of AF Burden: This domain analyzes the temporal pattern of AF episodes (paroxysmal, persistent, etc.), which influences treatment decisions.
- Substrate of AF: This domain assesses the presence of comorbidities, cardiovascular risk factors, and AM. Understanding these underlying factors is crucial for tailoring treatment strategies.

The 4S-AF characterization empowers clinicians to make informed decisions regarding anticoagulation and rhythm/rate control based on a patient’s specific profile.

To further assist clinicians in the management of AF patients, another score has been developed by Mariani et al. [153] that predicts spontaneous conversion to sinus rhythm in patients presenting to the emergency department with hemodynamically stable, symptomatic AF during a 6 h “wait and see” approach. The score takes into account previous history of spontaneous cardioversion (3 points), AF-related symptom duration < 24 h (5 points), age ≥ 65 years (3 points), and female sex (2 points) and is allowed to predict spontaneous conversion to sinus rhythm with good accuracy. 

These studies highlighted the complexity of AF management and the necessity to provide a tailored approach to every patient according to the burden of comorbidities, symptom severity, AF burden, and an individual patient’s specific risk, all of which might be reflected by the presence of AM and its severity. 

Current clinical guidelines on AF [1] advocate for the “Atrial Fibrillation Better Care (ABC)” pathway, which emphasizes a multi-pronged approach:

- Avoiding Stroke with Anticoagulation: This pillar focuses on preventing thromboembolic complications through appropriate anticoagulation therapy.
- Better Symptom Control: This pillar addresses patient symptoms by employing rate control or rhythm control strategies.
- Cardiovascular Risk Modification and concomitant diseases: This pillar aims to manage underlying cardiovascular risk factors, also through lifestyle modifications, to prevent disease progression and improve overall health outcomes.

The effectiveness of this integrated approach is evident in studies demonstrating a reduction in major adverse events, including stroke, in patients with AF [1].

While further research is necessary, accumulating evidence suggests potential benefits of early rhythm control (ERC) strategies in managing AF. Catheter ablation has emerged as a superior method compared to antiarrhythmic medications for maintaining sinus rhythm and avoiding medication-related side-effects. Notably, a population-based study linked AF ablation with a significantly lower incidence of dementia, HF, stroke, and mortality [24]. The EAST-AFNET 4 trial [154] demonstrated that systematic ERC improves clinical outcomes in all patients with AF, regardless of the presence of symptoms. A successive sub-analysis of the trial has demonstrated the presence of sinus rhythm at 12 months after randomization to explain most of the reduction in cardiovascular outcomes achieved by ERC [155], emphasizing the importance of an early and sustained restoration of sinus rhythm, while AF ablation was not associated with better outcomes compared with antiarrhythmic drug therapy. Dickow et al. [156] have replicated the clinical benefits of ERC strategies seen in the EAST-AFNET 4 trial in the general US population with newly diagnosed AF, suggesting the adoption of ERC as part of the management of patients with a recent diagnosis of AF in the US population. Further trials and investigations on AF management strategies may help to clarify the role of AF ablation and antiarrhythmic drug therapy for outcome reduction in these patients. 

Overall, these findings suggest that rhythm control strategies may offer broader health benefits beyond preventing stroke. However, it is crucial to acknowledge that restoring sinus rhythm does not address underlying risk factors. Conditions like hypertension, obesity, diabetes, and sleep apnea can contribute to AM, potentially hindering left atrial function even after successful rhythm control.

The “C” component of the ABC pathway emphasizes detection and management of cardiovascular risk factors, underlining the efficacy of lifestyle modifications. While the precise mechanisms linking these conditions to AF remain under investigation, they likely involve a pro-inflammatory state, leading to endothelial dysfunction and AM [157].

Managing these risk factors has demonstrably improved AF outcomes [158]. Studies have shown that lifestyle changes, including smoking cessation, alcohol abstinence, and regular exercise, significantly reduce the risk of dementia and stroke in individuals with AF [159,160,161].

The inclusion of AM assessment, along with monitoring its progression, could be a valuable addition to this structured approach. Evaluating AM could therefore serve as:

- a guide for anticoagulation strategies within the “A” pillar of the ABC pathway;
- a factor influencing the decision between rhythm/rate control strategies in the “B” pillar, as impaired atrial function may affect treatment efficacy [162];
- an early marker of cardiac disease or progression in patients with multiple cardiovascular risk factors within the “C” pillar.

By incorporating AM assessment into the current framework, clinicians can further personalize treatment plans and optimize patient outcomes in AF management.

Relying on these assumptions and on the strong emerging evidence, many authors started to consider AM not just as a “collateral finding” or a consequence of AF, but likely to be the primary cause of the morbidity and poor outcome previously attributed to AF (Table 3).

Sade et al. have demonstrated that a reduction in LA strain was able to stratify the risk of AF onset in patients with cryptogenic stroke irrespective of AF [163]. Moreover, in a low-risk general population, Alhakak et al. have shown a reduction in LA reservoir strain as an independent predictor of long-term risk for AF and ischemic stroke [164]. 

Azemi et al. have also demonstrated that in patients with low-risk CHA2DS2-VASc scores and a history of AF, LA strain values were significantly reduced in those presenting later with stroke or TIA compared with age- and gender-matched controls with identical CHA2DS2-VASc score [165], thus suggesting low LA strain as a direct contributor to thrombogenic risk (or both). Also, Saha et al. showed a strong association between impairment of LA strain, the presence of AF, and higher CHA2DS2-VASc score, and the fact that, in AF patients, LA strain was a predictor of stroke events and cardiovascular outcomes [166].

LA strain provided additional information on acute embolisms over and above the CHA2DS2-VASc score in a population of patients with paroxysmal or permanent AF [167]. Finally, LA strain has proved to be a predictor of new-onset AF in patients with HF [168] and those with cardiac amyloidosis, being able to identify those at high thrombotic risk, independent of AF [169].

Evaluation of LA strain, reflecting an underlying AM, also demonstrated a potential as a preoperative predictor of postoperative AF in patients undergoing aortic valve replacement (AVR) for aortic stenosis and coronary artery bypass grafting (CABG). Cameli et al. [170] demonstrated a significant reduction in global peak atrial longitudinal strain (PALS) as the sole independent predictor of postoperative AF. Pastore et al. [171] also proposed a preoperative global PALS below 28% as a specific parameter for stratifying patients at increased risk for postoperative AF (hazard ratio, 3.6 [95% CI, 2.2–5.9]; *p* < 0.001) following CABG. These findings emphasize the use of LA strain assessment in identifying patients with a higher risk of arrhythmias and applying optimum preventive measures.

A recent interesting attempt to demonstrate the possible influence of underlying AM in increasing the risk of ischemic stroke and dementia in patients with AF has been made in the study by Zhang et al. [172], in which the association of AF with stroke and between AF and dementia, adjusted by echocardiographic parameters of AM, has been investigated. The study was particularly powered by a large number of patients. The authors found that, after adjusting for LA volumes and function, particularly of LA reservoir strain, the apparent association between AF and incident stroke and dementia loses its strength and statistical significance. Building on this strong foundation, these studies demonstrated that LA strain analysis holds substantial promise. This method has shown not only to be able to identify LA fibrosis and early changes in the interstitial matrix, features of AM, but it has also showed to be an independent predictor of both new-onset AF and stroke risk, even in the absence of AF, being a predictor of cardiovascular outcomes. 

**Table 3:** Most relevant studies about the association between atrial myopathy and thromboembolic risk. LA, left atrial; TIA, transient ischemic attack; AF, atrial fibrillation; PALS, peak atrial longitudinal strain; AL, light chain amyloidosis; ATTR, transthyretin amyloidosis.

Most relevant Studies about the Association between Atrial Myopathy and Thromboembolic Risk				
Author/Year	Study Design	Study Setting	Number of Patients (n)	References
Sade et al. 2022	Prospective, single-center cohort study	LA strain to predict recurrence of TIA/stroke or new onset of AF in patients with no history of AF enrolled after a cryptogenic stroke event	186	[163]
Alhakak et al. 2022	Prospective, multi-center longitudinal study	General low-risk population long-term risk of AF and ischemic stroke based on baseline PALS	400	[164]
Azemi et al. 2012	Retrospective, single-center cohort study	Patients with AF, stroke, or TIA with CHA2DS2-VASc scores ≤ 1 before their events	57	[165]
Saha et al. 2011	Cross-sectional, single-center cohort study	Association between echocardiographic parameters and CHA2DS2-VASc score in patients with nonvalvular AF	77	[166]
Obokata et al. 2014	Prospective, observational, single-center cohort study	LA strain incremental value over CHA2DS2-VASc score in AF patients with or without acute embolism	286	[167]
Akintoye et al. 2023	Prospective, single-single center cohort study	LA strain prognostic value to predict incident thrombotic event in patients with AL or ATTR amyloid cardiomyopathy and no history of AF	448	[169]
Zhang et al. 2023	Prospective, multi-center study	Association of prevalent and incident AF with stroke and dementia adjusting for LA function and size.	5458 (stroke analysis) 5461 (dementia analysis)	[172]
Kamel et al. 2024	Randomized, double-blind, double dummy, multi-center study	Apixban 5 mg twice daily vs. aspirin 81 mg once daily in patients ≥ 45 years of age with embolic stroke of undetermined source, evidence of AM and no AF.	1015	[173]

The ARCADIA trial [173] marked a pioneering effort to introduce AM assessment into clinical practice as a guiding factor for anticoagulation therapy in patients with cryptogenic stroke and evidence of AM but no history of AF. The trial randomized patients to Apixaban 5 mg or 2.5 mg twice daily versus aspirin 81 mg once daily. The trial failed to demonstrate superiority in reducing recurrent stroke events in the anticoagulation group compared to the aspirin group (hazard ratio, 1.00 [95% CI, 0.64–1.55]). However, the criteria used in the study to define AM were based on the presence of ≥1 of:

- V1 P-wave terminal force >5000 µV·ms;
- serum N-terminal pro-B-type natriuretic peptide (NT-proBNP) >250 pg/mL;
- indexed LA diameter ≥3 cm/m^2^ on transthoracic echocardiography.

The inclusion of NT-proBNP > 250 pg/mL (elevated in the presence of left ventricular hypertrophy, renal failure, liver cirrhosis, pulmonary hypertension, etc.) among the AM criteria could have introduced a selection bias, leading to the inclusion of patients without true AM. This further highlights the ongoing need for a reliable method to assess AM in clinical practice.

The main limitations of the methodology are currently the absence of consensus or standardization for the echocardiographic definition of AM. All these authors have used strain parameters that, despite being a sensitive marker of atrial structural rearrangements and fibrosis, still remain “new indices” and are yet not regarded as an objective parameter for defining AM. A key element that can facilitate the standardization of the echocardiographic definition of AM is that the results of the main LA strain studies have not shown significant differences in calculated LA strain values between different vendors, nor any substantial gender-related differences. Instead, a negative association with increasing age has emerged, which could potentially justify age-related reference values [143,144].

**Figure 1:**
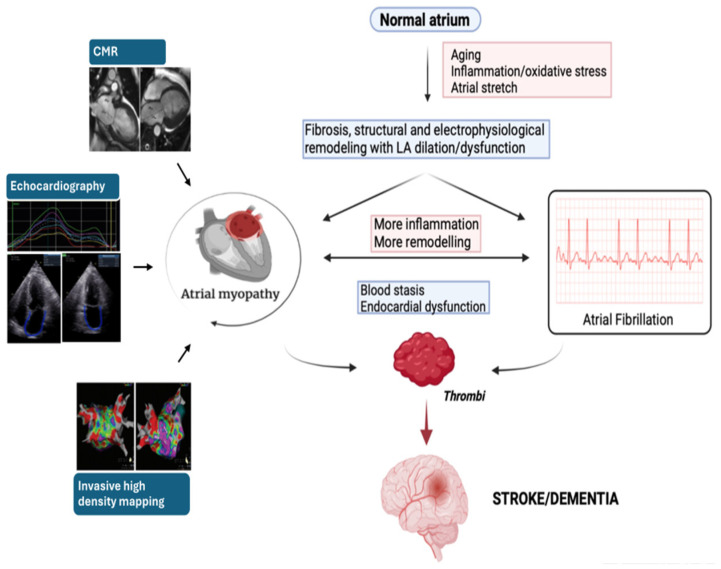
The interdependency and common pathophysiology of atrial myopathy and atrial fibrillation and their association with stroke and dementia. CMR, cardiac magnetic resonance.

LA strain has already been included as an additional parameter in detecting diastolic dysfunction in the latest recommendations of the European Association of CardioVascular Imaging (EACVI) on the use of multimodality imaging for the evaluation of HF with preserved ejection fraction (HFpEF) [174]. Thus, if the mechanism hypothesized by Zhang et al. for the association between AM and stroke (Figure 1), based on impaired LA structure/function predisposing to thrombus, is confirmed, the use of LA strain in evaluating AM may be considered for better studying the underlying mechanism and better stratifying a patient’s thromboembolic risk.

Considering the increasing availability, feasibility, and timelines of obtaining LA strain values, as well as being used within scores as a prognostic stratifier of cardiovascular outcomes, it may also be used to improve risk stratification for stroke and dementia, with important therapeutic consequences on anticoagulation for primary prevention of stroke. This may lead to a general optimization of anticoagulation use, not only including patients at higher risk of stroke without AF but also those in the grey zone risk of stroke according to current indices (i.e., in the low CHA2DS2-VASc score). These suggestions need randomized controlled trials, with more specific selection criteria for the definition of AM, and with safety/efficacy data on AM as a lone indication of anticoagulation, which are eagerly awaited in the future.

## 7. Conclusions

This article examines the emerging concept of AM, its association with thromboembolic events, and the consequent increased risk of stroke and dementia, particularly in the context of AF. While the association between AF and these outcomes is well known, AM presents a novel perspective, suggesting its possible role as an independent risk factor.

The key takeaway messages are three. (1) AM encompasses structural and functional abnormalities of the atria, potentially contributing to thromboembolism even in the absence of AF. (2) Echocardiography emerges as a promising tool for non-invasive evaluation of AM, particularly LA strain analysis. (3) Evidence exists suggesting that LA strain may offer additional risk stratification beyond the established CHA2DS2-VASc, with potential impacts on anticoagulation.

By acknowledging AM’s potential role and utilizing advanced imaging techniques like LA strain, we can strive towards a more comprehensive understanding of the mechanism underlying an increased thromboembolic risk in specific sub-groups of patients. The integration of LA strain analysis into routine clinical practice holds promise for improved patient management and targeted therapies, and thus may allow better clinical risk stratification in AF patients. Nevertheless, standardized definitions and diagnostic criteria for AM are crucial for consistent evaluation and research, and further studies are necessary to explore the efficacy and safety of anticoagulation in AM patients without AF.

## Figures and Tables

**Table 1:** The EHRA/HRS/APHRS/SOLAECE (EHRAS) classification of atrial myopathy [28].

Atrial Myopathy: EHRAS Classification	
***EHRAS class***	***Histological features***
***I***	Morphological/molecular changes affecting the cardiomyocytes (hypertrophy and myocytolysis). Absence of significant tissue fibrosis or interstitial changes
***II***	Predominance of fibrotic changes. Normal appearance of cardiomyocytes
***III***	Combination of changes in the cardiomyocyte and tissue fibrosis
***IV***	Non-fibrotic alteration of interstitial matrix
***a***	Amyloid accumulation
***f***	Fatty infiltration
***i***	Inflammatory cells
***o***	Other interstitial alterations

**Table 2:** Most relevant studies regarding LA strain values in healthy subjects. EACVI, European Association of Cardiovascular Imaging; IQR, interquartile range; NORRE, The Normal Reference Ranges for Echocardiography study; SD, standard deviation.

Most Relevant Studies Regarding LA Strain Values in Healthy Subjects				
Study Name/Author, Year	Study Design	Number of Patients (n)	Normal Pals Values (mean ± sd or Medial IQR)	References
Cameli et al., 2009	Obstervational, monocentric study	60	42.2% ± 6.1%	[124]
Pathan et al., 2016	Systematic review and meta-analysis	2542	39.4% (38.0–40.8%)	[144]
EACVI NORRE study, 2018	Observational, multicenter study	371	42.5% (36.1–48.0%)	[143]
